# Effect of Novel Quercetin Titanium Dioxide-Decorated Multi-Walled Carbon Nanotubes Nanocomposite on *Bacillus subtilis* Biofilm Development

**DOI:** 10.3390/ma11010157

**Published:** 2018-01-18

**Authors:** Diana S. Raie, Eisha Mhatre, Doaa S. El-Desouki, Ahmed Labena, Gamal El-Ghannam, Laila A. Farahat, Tareq Youssef, Wolfgang Fritzsche, Ákos T. Kovács

**Affiliations:** 1 Process Design and Development Department, Egyptian Petroleum Research Institute (EPRI), Nasr City 11727, Cairo, Egypt; doaadesouki@hotmail.com (D.S.E.-D.); labena.labena@gmail.com (A.L.); lailafarahat@yahoo.com (L.A.F.); 2 Terrestrial Biofilms Group, Institute of Microbiology, Friedrich Schiller University Jena (FSU), Jena 07743, Germany; eisha.mhatre@jsmc.info; 3 National Institute of Laser Enhanced Sciences (NILES), Cairo University, Giza 12613, Egypt; Gamalniles@cu.edu.eg (G.E.-G.); tareq.youssef@niles.edu.eg (T.Y.); 4 Nanobiophotonic Department, Leibniz Institute of Photonic Technology Jena (IPHT), Jena 07745, Germany; wolfgang.fritzsche@leibniz-ipht.de; 5 Bacterial Interactions and Evolution Group, Department of Biotechnology and Biomedicine, Technical University of Denmark, Kgs. Lyngby 2800, Denmark

**Keywords:** titanium oxide nanoparticles, quercetin, multi-walled carbon nanotube, bacterial adhesion, biofilm, hydrophilicity, bacillus subtilis

## Abstract

The present work was targeted to design a surface against cell seeding and adhering of bacteria, *Bacillus subtilis*. A multi-walled carbon nanotube/titanium dioxide nano-power was produced via simple mixing of carbon nanotube and titanium dioxide nanoparticles during the sol-gel process followed by heat treatment. Successfully, quercetin was immobilized on the nanocomposite via physical adsorption to form a quercetin/multi-walled carbon nanotube/titanium dioxide nanocomposite. The adhesion of bacteria on the coated-slides was verified after 24 h using confocal laser-scanning microscopy. Results indicated that the quercetin/multi-walled carbon nanotube/titanium dioxide nanocomposite had more negativity and higher recovery by glass surfaces than its counterpart. Moreover, coating surfaces with the quercetin-modified nanocomposite lowered both hydrophilicity and surface-attached bacteria compared to surfaces coated with the multi-walled carbon nanotubes/titanium dioxide nanocomposite.

## 1. Introduction

Designing surfaces for controlling biofilm development has become an important scope for both detrimental and beneficial biofilm technologies. Biofilms are surface-associated microbial communities encased in a self-produced extracellular polymeric substance (EPS) [1]. They can develop everywhere on almost all natural materials [2] like rock [3], sand [4], soil [5], skin, teeth, plants, etc. Also, artificial surfaces [6,7] such as plastics [8], glasses [9] and metals [10] are suitable substrata for biofilm formation. In water and wastewater treatment facilities, they are causative agents for corrosion [11] and contamination [12]. In addition, they reduce efficiency of heat exchangers [13]. On ship hulls, marine fouling results from the aggregation of microbial biofilms and larger marine organisms, can upsurge the fuel cost of seafaring vessels [14]. Besides these dramatic impacts, biofilms have an increased resistance to antimicrobial agents compared to plankton counterparts. The biofilm architecture could contribute to resistance by exclusion of biocides from the bacterial community. They can also develop biocide-resistant phenotypes due to the heterogeneity of the single biofilm community [15]. Therefore, on one hand, intensive research activities were directed to develop techniques for overcoming microbial biofilm-related problems in most industrial systems. On another hand, biofilms have useful aspects in various environmental applications. The surface-attached biomass can degrade organic materials or adsorb heavy metals [16] quicker than activated sludge [16,17]. In addition, biofilms have a higher stability towards the toxic pollutants and variable conditions due to EPS. The smaller volume of biomass and the lower economic value of the wastewater treatment process are added value of applying biofilms in such sectors [18]. Furthermore, certain bacteria can transfer electrons from the microbial cell to an electrode or vice versa instead of a natural redox partner. These electroactive bacteria can form electroactive biofilms on conductive materials resulting in a direct electrochemical connection with the electrode surface while using it as electron exchanger without the aid of mediators. Therefore, these biofilms are used in microbial fuel cells (MFCs) for generating energy via treating wastewater [19]. Basically, in MFCs, bacteria adhere to the surface of the anode (the negative electrode) forming biofilms. Such a bio-electrode acts as a catalyst to convert the chemical energy of the organic molecule into electrons [19,20,21,22]. Therefore, optimizing the surface and microbial interaction is one of the most effective factors for inhibiting or inducing biofilms and, in turn, many of their applications. Generally, bacterial adhesion to a substratum is controlled by different factors including attraction, i.e., van der Waals forces, repulsion, i.e., steric interactions and electrostatic forces, and thermodynamics, i.e., hydrophobic/hydrophilic and osmotic interactions [23]. Therefore, bacterial surface-adhesion and subsequent biofilm formation is controlled by surface topography [23]. Indeed, carbon nanotubes (CNTs) are anode materials that express anti-corrosive activity, biocompatibility and chemical and microbial stability. Moreover, they have high conductivity, surface area, mechanical strength and toughness. However, their nanoscale topology was described to frustrate bacterial adhesion due to reducing the contact area [24] and inducing repulsive forces for bacterial cells [25]. Besides, the antimicrobial and anti-biofilm activity of CNTs can prevent bacterial adhesion. The chemical decoration of CNTs by titanium oxide nanoparticles (TiO_2_ NPs) [9] can improve their extraordinary electrical, mechanical, thermal properties and wettability conversion behavior of CNTs/TiO_2_ [26]. Particularly, CNTs were reported to create a special confinement of TiO_2_ and large supporting surface areas, leading to faster reaction rates. Although TiO_2_ NPs showed no anti-microbial activity in dark condition [27], they promote anti-adhesive efficiency against bacteria [9]. Therefore, immobilized biomolecules in tailor-made nanoscale architectures can significantly advance their behavior [28]. Quercetin (Q), a widely distributed flavonoid, forms H-bonds that increase its surface adsorption properties [29]. Likewise, Q was reported to be thermally stable [9], electro-chemically active [30] and biologically safe [31]. Also, it provided a suitable surface for bacterial adhesion [9]. Because most of natural biofilms are mixed microbial communities, single-species biofilms were used as model bacteria. Over the past decade, *Bacillus subtilis* has been considered the bacterium of choice to be the studied as a Gram-positive model of beneficial biofilm applications. In addition to being non-pathogenic, *B. subtilis* cells are capable of forming dormant spores that are resistant to extreme conditions, and thus, can be easily formulated and stored [32]. Therefore, the aim of the present work was directed to study the effect of nano-coating using titanium dioxide-decorated multi-walled carbon nanotubes (MWCNTs/TiO_2_) and its Q-modified nanocomposite quercetin/titanium dioxide-decorated multi-walled carbon nanotubes (Q/MWCNTs/TiO_2_), in respect to their wettability, on the microbial adhesion of *B. subtilis*. To our knowledge, this study can be considered the first to prepare and report the proliferative activity of quercetin/multi-walled carbon nanotube/titanium dioxide nanocomposite for bacteria.

## 2. Results

**Figure 1:**
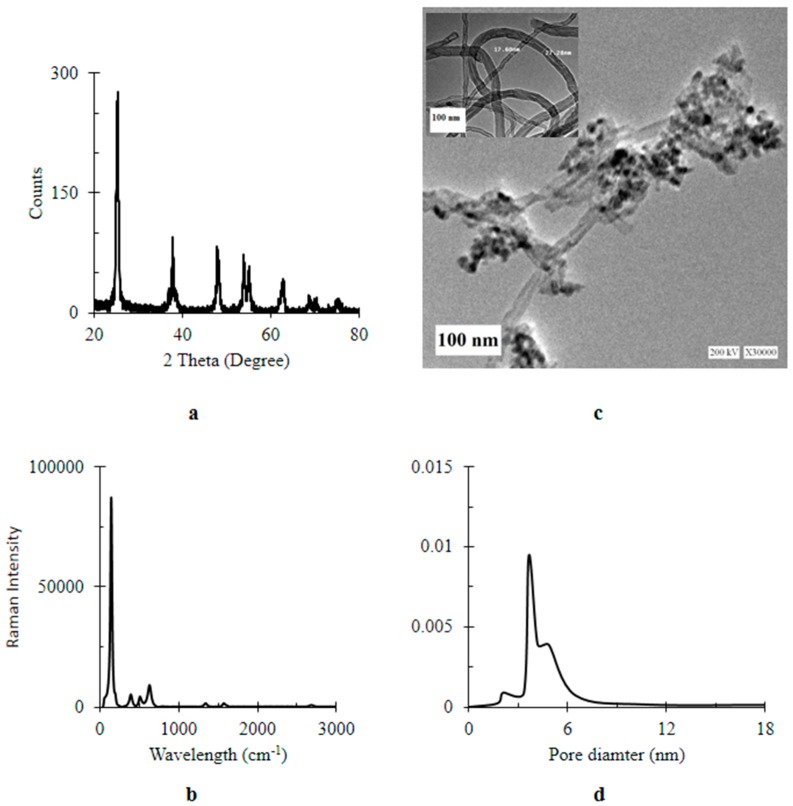
Characterization of titanium dioxide-decorated multi-walled carbon nanotubes (MWCNTs/TiO_2_) (**a**) XRD (X-Ray Diffraction) pattern; (**b**) Raman spectrum; (**c**) TEM (Transmission Electron Microscopy) image at a magnification bar of 100 nm (inset bare MCNTs); (**d**) pore diameter.

Morphological and spectroscopic characterization of the prepared MWCNTs/TiO_2_ are illustrated in Figure 1. In Figure 1a, the XRD diffractogram showed sharp intense peaks at 25.4°, 36.1°, 37.8°, 48°, 54.3°, 55.3°, 63°, 69° and 70° for diffraction from {101}, {103}, {004}, {200}, {005}, {105}, {211}, {204}, {116} and {220} planes; respectively, indicating a high crystalline pure tetragonal anatase phase for TiO_2_ NPs (JCPDS 01-071-1167). Based on Scherrer’s equation, the crystal size of TiO_2_ was 9.85 nm. Additional two peaks at 26.0° and 43.6° were attributed to {002} and {100} facets of hexagonal graphite structure of MWCNTs [33] of calculated crystal size 36.17 nm. According to Wen et al. [34], the number of walls of MWCNTs was estimated to be 11. Interestingly, TiO_2_ peak at 25.4° was overlapping the main peak of MWCNTs at 26 was observed [34]. The oxide form of titanium was formed as a result of supplying titanium precursor; TTIP, by the alcoholic solvent, i-PrOH. Besides, anatase formation was developed by a gradual structural rearrangement of the titanium–oxygen lattice of the amorphous reactants, and thermal treatment. In addition to the slow reaction rate, an organic species was formed and acted as a capping agents controlling the crystal growth and influenced particle morphology as well as assembly behavior [35].

After calcination, these organic stabilizers were decomposed [9] as confirmatively displayed by Raman spectrum (Figure 1b). The structural ordering of the nanocomposite was additionally analyzed by Raman Spectroscopy. Five peaks appeared at 143.8 (Eg), 198.0 (Eg), 395.7 (B_1g_), 516.6 (A_1g_ + B_1g_) and 637.6 (Eg) cm^−1^ which corresponds to the symmetric active modes of the anatase phase of the prepared TiO_2_ NPs. Moreover, two extra distinct bands at 1347 cm^−1^ and 1575 cm^−1^ were known as D band and G band corresponding to the presence of defect sites and the integrity of hexagonal carbon; respectively. The ratio of D band to G band (I_D_:I_G_ ≈ 1) indicated the presence of defective walls of CNTs. Furthermore, a weak shoulder appearing at 1600 cm^−1^ (G+ band) was also associated with the defects in the MWCNTs [34]. Figure 1c shows a TEM image illustrated the multi-walled carbon nanotubes (MWCNTs) with diameters ranging from 20 to 25 nm with a random aggregation of TiO_2_ particles on CNTs. The defects in the CNT walls can be clearly seen which make the contact between TiO_2_ and CNTs easier. These defects appeared after functionalization of MWCNTs by TiO_2_NPs as shown in the inset of Figure 1c. This TEM image confirmed the purity of the prepared bare MWCNTs as predicted by the Raman spectrum (see Figure 1b). The controlled growth of the crystal size during the drop-wised reaction in addition to the capping effect of the organic species led to uniform particle morphology [35] as presented by the TEM image (Figure 1c), which shows an irregular spherical shape NPs within a size range of 35–60 nm. Notably, the observed aggregated NPs were attributed to the attractive van der Walls forces [36]. Therefore, a large exposed surface area was predicted for MWCNTs/TiO_2_ due to their nanosize. The mean external surface area of MWCNTs/TiO_2_ was estimated to be 137 m^2^ g^−1^. The pore size was in a range of 2–50 nm and the cumulative pore volume was 0.45 cc g^−1^ (Figure 1d). Convincingly, the present MWCNTs/TiO_2_ was described as a mesoporous material [37].

**Figure 2:**
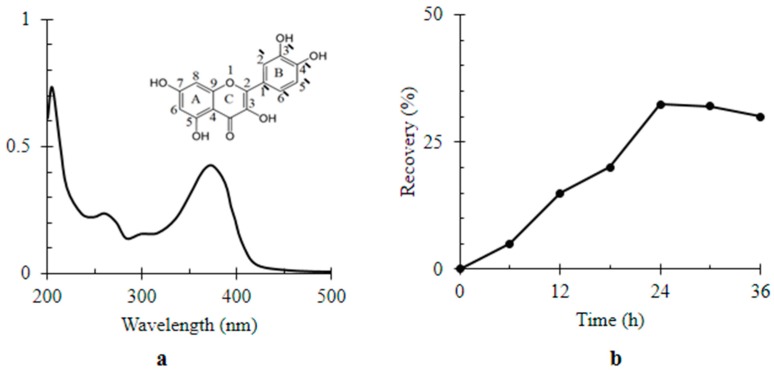
Colorimetric assay of quercetin (Q) (**a**) UV-visible spectrum of Q [1 × 10^−6^ M]. Inset the chemical structure of Q; (**b**) the rate of Q relative recovery by titanium dioxide-decorated multi-walled carbon nanotubes (MWCNTs/TiO_2_) over time (36 h).

Optically, Q showed absorbance in the UV-visible region with three identified peaks in its spectrum at λ_204_, λ_258_ and λ_374_ nm (Figure 2a). According to the Beer–Lambert’s law, the rate of Q relative recovery percentage by MWCNTs/TiO_2_ over 36 h was represented by Figure 2b. The relative recovery of Q by MWCNTs/TiO_2_ gradually increased to reach its maximum value i.e., 32.45 ± 0.93% of the dissolved Q after 24 h of incubation time. Definitely, the potentiality of MWCNTs/TiO_2_ to adsorb Q was attributed to both the large surface area and its special surface characteristics [38]. The abundant active sites of CNTs like unsaturated suspending bonds, pentagons carbon loops, and pentagon–heptagon defect pairs can react with some hydroxyl groups of Q (see inset Figure 2a) resulting in adsorptions. Besides, Q energetically favored accumulation and assembly on the MWCNT interface due to being insoluble in water, with a strong hydrophobic property and high interfacial activity [38].

**Figure 3:**
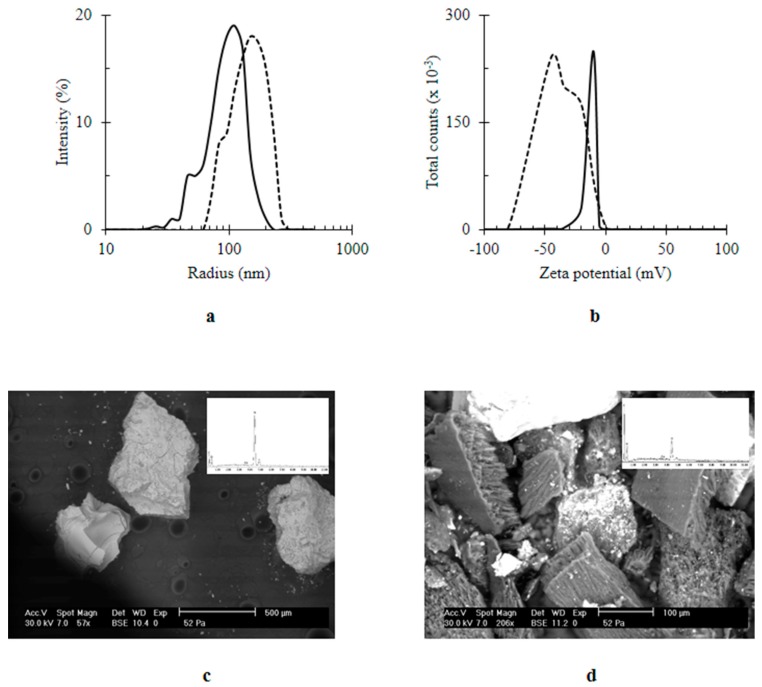
The effect of immobilization of quercetin (Q) on titanium dioxide-decorated multi-walled carbon nanotubes (MWCNTs/TiO_2_) (**a**) size (nm); (**b**) zeta potential (mV); (**c**) SEM image of MWCNTs/TiO_2_; (**d**) SEM image of Q/MWCNTs/TiO_2_. In (**a**,**b**) MWCNTs/TiO_2_ was represented by the continuous line (ـــــــــ) and the quercetin titanium dioxide-decorated multi-walled carbon nanotubes nanocomposite (Q/MWCNTs/TiO_2_) was denoted by the dashed line (----). Inset (**c**,**d**) EDX spectra.

After introducing Q, there was an increase in the size of nanocomposites from 155.33 ± 32.49 to 200 ± 69.3 nm; Q/MWCNTs/TiO_2_ was quantified (Figure 3a). The negative charge of the MWCNTs/TiO_2_; −8.5 ± 2.95 mV (Figure 3b) was generated from adsorption of water’s hydroxyl groups on the surface of the nanocomposite [39]. However, in the presence of Q, the negativity of the nanocomposite upturned into −45.0 ± 20.4 mV (Figure 3b). Likewise other biogenic polyphenols [40], the improved negativity of Q-based nanocomposite was attributed to five hydroxyl groups of Q [9] (see the chemical composition of Q inset Figure 2a). Furthermore, the immobilization of Q on MWCNTs/TiO_2_ was confirmed by SEM analysis (Figure 3c,d). The higher intensity of the carbon peak revealed by EDX (inset Figure 3d) was an indicator for the content of Q in the Q-owned nanocomposite (inset Figure 2a).

**Figure 4:**
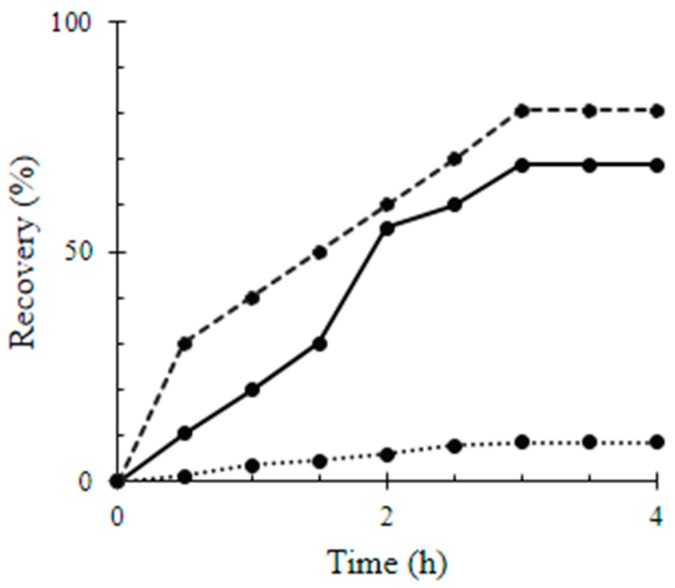
Relative recovery percentage of quercetin (Q), titanium dioxide-decorated multi-walled carbon nanotubes (MWCNTs/TiO_2_), and quercetin titanium dioxide-decorated multi-walled carbon nanotubes nanocomposite (Q/MWCNTs/TiO_2_) by the glass slides over time. Q, MWCNTs/TiO_2_ and Q/MWCNTs/TiO_2_ was symbolized by the dot (…..), continuous (ـــــــــ) and the dashed (----) lines; repetitively.

Gravimetrically, the relative recovery rates of Q, MWCNTs/TiO_2_, and Q/MWCNTs/TiO_2_ by the glass slides over 4 h are represented by Figure 4. The surface coverage of Q on the glass slides was 27.26 ± 0.22 mg mm^−2^; i.e., the adsorption capacity of the glass to Q was 8.45 ± 0.72% (Table 1).

**Figure 5:**
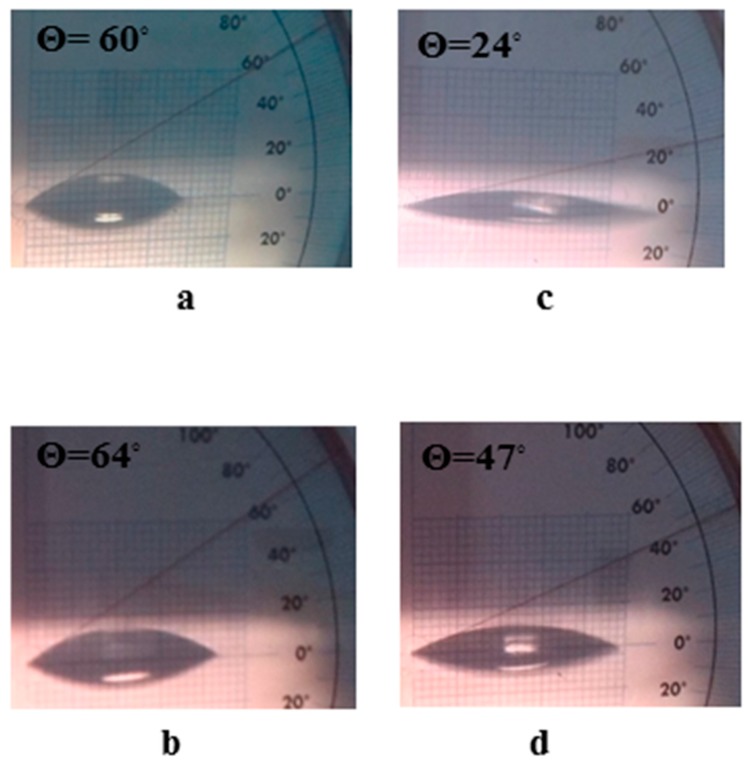
Optical images for water contact angle (θ) to glass surfaces that were uncoated (**a**) or coated by quercetin (Q) (**b**), titanium dioxide-decorated multi-walled carbon nanotubes (MWCNTs/TiO_2_) (**c**) quercetin quercetin titanium dioxide-decorated multi-walled carbon nanotubes nanocomposite (Q/MWCNTs/TiO_2_) (**d**).

The glass slides adsorbed and recovered 68.75 ± 0.01% of the total suspended amount of MWCNTs/TiO_2_ adsorbate, producing a surface coverage 33.03 ± 0.07 × 10^−2^ mg mm^−2^ (Table 1). However, the improved efficiency in Q/MWCNTs/TiO_2_ recovery by glass into 80.63 ± 0.38% to produce surface coverage of 35.63 ± 1.13 × 10^−2^ mg mm^−2^ (Table 1) was a result of the multiple opportunities of forming H-bonds required for adsorption [29]. Moreover, the water contact angle of untreated and Q-coated glass surfaces was changed from 60° and 64° (a hydrophilic feature) to 24° and 47° (an improved hydrophilic behavior) after coating by MWCNTs/TiO_2_ and Q/MWCNTs/TiO_2_ (Figure 4a–d), respectively. The wettability behavior of the uncoated slides was attributed to the tendency of borosilicate glass to adsorb water owing to their Si-OH group terminated polar surfaces [41]. However, losing three-fifths of the contact angle value after coverage of the surface by MWCNTs/TiO_2_ was related to the interaction of water with CNTs. Briefly, the steep decrease in contact angle resulted from water condensation inside the CVD tubes based on the superior curvature of the inner interface of nanotubes and on their surfaces eventually in the space between the tube and the surface holder [42]. The reported hydrophobicity of Q molecules [43] can contribute to the decrease in the surface hydrophilicity (see Figure 5b).

**Figure 6:**
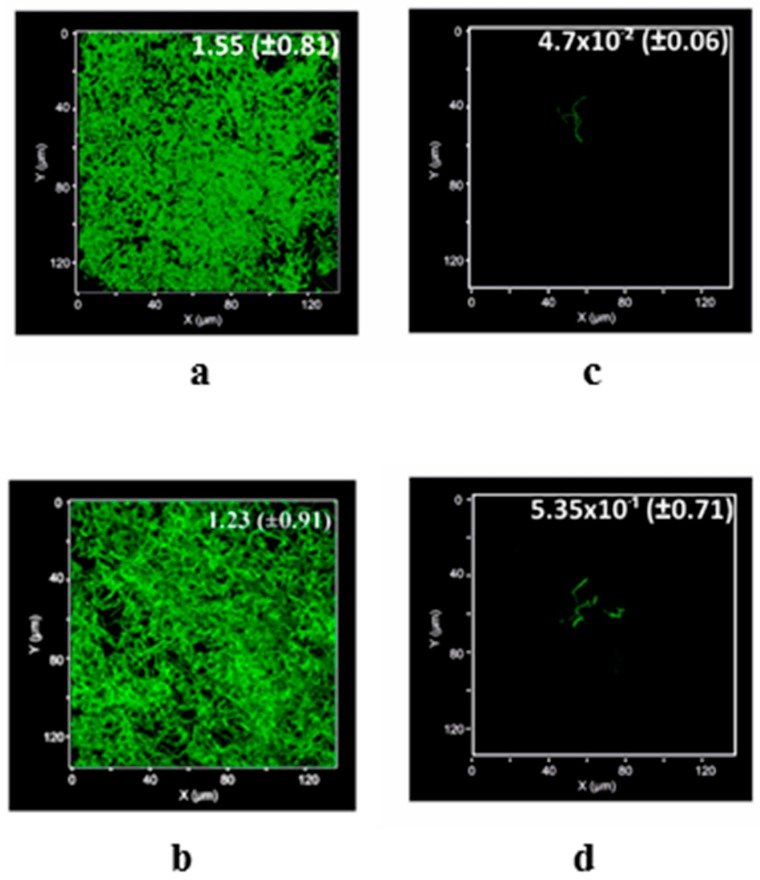
Mean biovolume of *B. subtilis* (µm^3^ µm^−2^) developed on the uncoated glass surfaces (**a**) or coated by quercetin (Q) (**b**), titanium dioxide-decorated multi-walled carbon nanotubes (MWCNTs/TiO_2_) (**c**), and quercetin titanium dioxide-decorated multi-walled carbon nanotubes (Q/MWCNTs/TiO_2_) (**d**).

Nevertheless, because of the reported negative charges of the uncoated glass slide [41], the bacterial cells preferred to attach to the surface rather than the surrounding liquid medium (Figure 6a) with an estimated biovolume of 155 × 10^−2^ ± 81.0 µm^3^ µm^−2^ after 24 h of the incubation. Indeed, *B. subtilis* as a Gram-positive model bacteria had a high degree of hydrophobicity [44] and a great potency for adhesion. Therefore, the favorable attachment of bacillus cells to the glass surface was attributed to forming hydrogen bonding [45]. Successfully, *B. subtilis* can develop on the surface coated by Q with a biovolume of 133 × 10^−2^ ± 0.91 µm^3^ µm^−2^ (Figure 6b); i.e., biovolume recovery of 85.81 ± 0.58%. However, the surface coated by TiO_2_/MWCNTs exhibited a great reduction in the biovolume of attached cells; 4.7 × 10^−2^ ± 0.06 µm^3^ µm^−2^ (Figure 6c) to produce levels of development that reached 3.03 ± 0.04% after 24 h. Initially, this anti-adhesion was attributed to the electrostatic repulsion [9] between the negatively charged TiO_2_/MWCNTs (see Figure 3b) and the negatively charged bacterial cells leading to weaken the cellular contact with the negatively charged substratum [9,46]. Moreover, the converse correlation between the bacterial cell attachment of micro-sized cells to nano-scaled surface (see Figure 3a) [46,47] can be contributing to such protection as the opportunity of cells to lay out between the narrow distance between nanoparticles was reduced [46]. Furthermore due to the porosity of TiO_2_/MWCNTs (see Figure 1d), the bacterial attachment to surfaces was minimized by small-diameter nanoscale pores [24]. Besides, the high hydrophilicity of the TiO_2_/MWCNTs coated surface (see Figure 5b) can efficiently diminish or inhibit bacterial adhesion as a result of the barrier hydration layer formed on the surface. This was resulted from the surface ability to take up enormous quantities of free water through both ion solvation and hydrogen bonding interaction [48]. Curiously, after 24 h of incubation, the surface coated by Q/TiO_2_/MWCNTs allowed the biofilm development of laboratory strain *B. subtilis* 168 with a slightly reduced bio-volume of 53.5 × 10^−2^ ± 0.71 µm^3^ µm^−2^ (Figure 5d) of which the recovery level was 34.52 ± 1.01%. Likewise, the repulsion between negative charges (see Figure 3b) enabled Q/TiO_2_/MWCNTs to save the substratum from bacterial physical attachment [9,46]. In addition, the relative decrease in attached biovolume of bacteria to the coated surface by Q/TiO_2_/MWCNTs was attributed to the improved hydrophilicity of its surface (see Figure 5c), and the inverse relationship between the bacterial attachment of micro-sized bacteria to nano-scaled coated surfaces (see Figure 3a) [47]. However a superior value of the charged Q/TiO_2_/MWCNTs in comparison to its counterpart was observed, with the former protection being less effective than the later. Actually, the duplication of water contact angle value of the covered surface by Q/TiO_2_/MWCNTs reduced its hydrophilicity (see Figure 5d) which had a negative effect on the protection level of the substratum.

## 3. Discussion

Overall, there was not an approximate potential growth of the bacterial cells on the coated surface by TiO_2_/MWCNTs. So, such surface can be used in industries where microbes induce dangerous effects like in food, pharmaceuticals, etc. Compared to dark conditions, coating glass slides with a negatively charged TiO_2_ (either anatase or amorphous) showed a repulsive activity against physical attachment of *B. subtilis* to the surface. Since most bacterial genera are negatively charged, the electrostatic repulsion weakened the cellular contact with the negatively charged substratum [9]. These nanocrystals can minimize the contact area between the bacterium and the surface leading to a reduction of bacterial adhesion [46]. However, quercetin had an impact on zeta potential and adsorption capacity of crystalline metal oxides; other experiments showed that the presence of Q has a meaningful impact on the ability of *B. subtilis* to develop a biofilm and consequently the application of the prepared materials. Interestingly, in comparison to the biomass growth on the uncoated glass, around two-thirds of the *B. subtilis* biovolume can develop on the coated surface by Q/TiO_2_/MWCNTs. Certainly, quercetin 2,3-dioxygenase was reported to be secreted by *B. subtilis*. This enzyme was described to be involved in the resistance mechanism of this bacteria. In addition, Q with its low water solubility, can provide opportunities for bacterial development. Such conductive bio-inspired nanocomposites in the presence of surface attached bacteria has a promising applicability in bio-electrochemical systems. Particularly, *B. subtilis* can generate electricity [49] and grow anaerobically [50]. Hence, examining the efficiency of such bio-electrodes to generate bio-energy will be an exciting prospective step.

## 4. Materials and Methods

### 4.1. Preparation of Nanomaterials

A composite of TiO_2_ nanoparticles and multi-walled carbon nanotubes (MWCNTs/TiO_2_) was prepared via sol-gel method [51]. Briefly, a 0.03 g of purified MWCNTs (EPRI) was added to a mixture of titanium (IV) isopropoxide (TTIP, Aldrich, St. Louis, MO, USA) and acidified isopropanol (i-PrOH, Fisher, Hampton, NH, USA) within a ratio 4:5 and pH range ~2. The mixture was sonicated for 40 min followed by adding a diluted i-PrOH under vigorous stirring for 30 min at 25 °C. Stirring for the resulting colloidal solution continued for 1 h until sol was developed, followed by aging overnight to form the corresponding gel. The sample was dried at 120 °C in oven and annealed at 450 °C to induce the phase transformation to crystalline anatase TiO_2_ while retaining the structural integrity of the CNTs [52]. Remarkably, the multi-walled carbon nanotubes (MWCNTs), have been synthesized via the Catalytic Chemical Vapor Deposition (CCVD) process [33]. To avoid nanoparticles agglomeration, MWCNTs were purified before through washing by strong acid (37% HCL, Aldrich). Furthermore, Q was immobilized on MWCNTs/TiO_2_ by dispersing a known weight of MWCNTs/TiO_2_ in the Q [1 × 10^−3^ M] solution under sonication for 5 min followed by shaking (100 rpm) at room temperature over a time range stating from 3 h to 36 h. After the incubation time, Q/MWCNTs/TiO_2_ was collected by centrifugation and washed 3 times by water then left for drying at 100 °C.

### 4.2. Characterization of Nanomaterials

The phases of prepared materials were identified by powder X-ray Diffraction (XRD) analysis using Analytical X’PERT PROMPD X-ray diffractometer, CuKα radiation of wavelength λ = 0.15406 nm, rating of 40 KV, 40 mA, step size = 0.02 and scan step time of 0.4 s in the 2θ range 20–80. The crystalline phases were matched to Joint Committee on Powder Diffraction Standards (JCPDS). The crystal size can be estimated using Debye–Scherrer’s equation:

(1) $$D_{x}=\frac{0.9\lambda }{\beta \cos\theta }$$

where; D symbolizes the average crystallite size (nm) of x; TiO_2_ or MWCNTs, β is the width of the peak at half maximum intensity (FWHM) of the main peak of anatase phase in radians and λ is the X-ray wavelength (λ = 0.154056 nm). The number of graphitic wall (N) of MWCNTs is calculated by the following relation [53]:

(2) $$N=\frac{D_{\mathrm{MWCNTs}}}{d_{\left\{002\right\}}}$$

The characteristic spectra of MWCNTs/TiO_2_ were recognized by the dispersive Raman Microscopy (Model Sentera, Bruker, Germany) of power l0 mW at laser wavelength of 532 nm (doubled Nd:YAG; Neodymium-doped Ytrium Aluminum Garnet, Laser). MWCNTs/TiO_2_ was checked using Transmission Electron Microscopy (TEM; JEOL JEM-2000EX Tokyo, Japan) to detect their morphology. The surface area of the MWCNTs/TiO_2_ was measured from nitrogen adsorption-desorption isotherms at liquid nitrogen temperature (77 K) using a Quantachrome AS1Win version 2.01 instrument. The samples were out-gassed for 3 h at 150 °C. The Brunauer–Emmett–Teller (BET) method was used for surface area calculation, while pore size distribution (pore diameter and volume) were determined by the Barrett–Joyner–Halenda (BJH) method. The concentration of adsorbed Q by MWCNTs/TiO_2_ (c) was characterized over time (36 h) optically using UV-Visible spectrophotometry applying Beer–Lambert’s law at λ_258_ nm for the absorbance (A), the molar absorptivity of Q (ε) is 562.341 cm^2^ mol^−1^ [54] and a path length of the sample (b) is 1 cm according to the relation:

(3) A= ε b c

The relative adsorption of Q by MWCNTs/TiO_2_ was expressed as a percentage as follow:

(4) $$\mathrm{Adsprption}\%=\frac{A}{A_{0}}\times 100$$

where A_0_ is the absorbance of Q at 0 time. The hydrodynamic diameter and zeta potential of the prepared nanomaterials were identified by dynamic light scattering spectroscopy (DLS; Malvern Instruments Ltd., Worcestershire, UK). The functionalization of MWCNTs/TiO_2_ by Q was tested by scanning electron microscopy (SEM, HITACHI, Tarrytown, NY, USA) combined by Energy Dispersive X-ray detector (EDX, Bruker, Madison, WI, USA).

### 4.3. Coating Glass Surface and Characterization

Under sonication for 10 min, the glass cover-slides (22 mm × 22 mm × 0.17 mm) were cleaned by triplicate washing with deionized water, ethanol, and acetone. After drying, the polished slides were dipped in an aqueous suspension of tested materials [1 × 10^−3^ M] for 3 h. The coverslips were dried in an oven at 100 °C. The mass of coating NPs (m) was estimated gravimetrically [55] as:

(5) $$m\left(\mathrm{mg}\right)=m^{\prime \prime }-m^{\prime }$$

where; m′ and m″ are the mass of the cleaned glass slide before and after immersing in the tested suspension; respectively. The adsorption capacity was quantified using the following equation:

(6) $$\mathrm{Adsorption}\;\mathrm{capicity}\%=\left(1-\frac{m\times 10^{3}}{\mathrm{Mwt}}\right)\times 100$$

Mwt. is the molecular weight of the tested nanomaterials. The recovery rate of the tested materials by the glass slide were characterized over time (4 h). Surface coverage (𝛤) expressed in mg mm^−2^ was quantified applying the following relation [55]:

(7) $$\Gamma =\frac{m}{S}$$

where, S is the total surface area of glass slide. Measuring the static contact angle of distilled water; JT Baker, HPLC grade, (θ) to the coated surfaces was performed applying the sessile drop technique using a Tantec line of contact angle meter apparatus (Germany).

### 4.4. Induced Biofilm Development Test

Bacteria cultivation: Overnight LB (Lysogeny Broth, LB-Lennox, 10 g L^−1^ tryptone, 5 g L^−1^ yeast extract, 5 g L^−1^ NaCl, pH 7.0, Carl Roth, Germany) grown culture of green fluorescently labelled *B. subtilis* 168 [56], was used as an inoculum (1% in 4 mL media) for biofilm growth medium (BGM; LB supplemented with 0.15 mol L^−1^ (NH_4_)_2_SO_4_, 100 mmol L^−1^ K_x_H_y_PO_4_ (pH 7.0), 34 mmol L^−1^ Na-citrate, 1 mmol L^−1^ MgSO_4_, 0.1% glucose, and 0.1 mmol L^−1^ MnCl_2_) in polystyrene petri dishes containing treated glass cover slides as described previously [9]. The plates were incubated at 30 °C in a static condition. Every 12 h, the medium was changed and replaced by fresh BGM in order to select specifically for the adhered cells [57].

Microscopy: After 24 h, the tested slides were collected from the petri dishes, placed on microscopy glass slides and then covered with coverslips (24 × 50 mm). A layer of nail polish was applied on the corner of the coverslips in order to fix this arrangement. The slides were then observed using Zeiss LSM780 Confocal Laser-Scanning Microscopy (CLSM) fitted with a 488-nm laser an EC Plan-Neofluar 63× oil immersion objective (Carl Zeiss Microscopy GmbH, Jena, Germany). About 6–10 independent locations were selected and recorded at random positions on the glass slides from independent duplicated samples. The image stacks were analyzed using Comstat script [58] and the biomass of the attached cells was estimated from the calculated intensities.

Percentage of biovolume recovery: The percentage of biovolume recovery of *B. subtilis* on a coated surface, was evaluated based on the ratio between the biovolume developed by *B. subtilis* on coated surface (C) by nano-agent (x; Q, MWCNTs/TiO_2_ or Q/MWCNTs/TiO_2_) and uncoated glass slide (U) by the previously used equation [9]:

(8) $$\mathrm{Biovolume}\;\mathrm{recovery}\;\left(\%\right)=\frac{C_{x}}{U}\times 100$$

## 5. Conclusions

In summary, glass surfaces were coated with titanium dioxide-decorated multi-walled carbon nanotubes and quercetin titanium dioxide-decorated multi-walled carbon nanotubes nanocomposites to study the behavior of *Bacillus subtilis* biofilm development. Our results indicated that coating surfaces with titanium dioxide-decorated multi-walled carbon nanotubes changed the hydrophilicity of uncoated surfaces into super hydrophilicity and, in turn, protected the surface from bacterial adhesion. However, coating surfaces with quercetin titanium dioxide-decorated multi-walled carbon nanotubes nanocomposites improved the hydrophilicity of the glass surface to some extent and increased the relative efficiency of bacterial adhesion. The production of such bio-inspired conductive mesoporous nanomaterials is important for electrode applications such as microbial fuel cells.

## Figures and Tables

**Table 1:** Characterization of glass surface after coating by quercetin (Q), titanium dioxide-decorated multi-walled carbon nanotubes (MWCNTs/TiO_2_), and quercetin titanium dioxide-decorated multi-walled carbon nanotubes nanocomposite (Q/MWCNTs/TiO_2_). (Mean value ± stranded deviation).

Material	Surface Coverage (mg mm^−2^)	Recovery Percentage (%)
Q	27.26 ± 0.22	8.45 ± 0.72
MWCNTs/TiO_2_	30.33 ± 0.07 × 10^−2^	68.75 ± 0.01
Q/MWCNTs/TiO_2_	35.63 ± 1.13 × 10^−2^	80.63 ± 0.38
